# Impact of *CFTR* Modulators on the Impaired Function of Phagocytes in Cystic Fibrosis Lung Disease

**DOI:** 10.3390/ijms232012421

**Published:** 2022-10-17

**Authors:** Aniello Meoli, Olaf Eickmeier, Giovanna Pisi, Valentina Fainardi, Stefan Zielen, Susanna Esposito

**Affiliations:** 1 Pediatric Clinic, Department of Medicine and Surgery, University of Parma, 43126 Parma, Italy; 2 Division of Allergy, Pulmonology and Cystic Fibrosis, Department for Children and Adolescents, University Hospital, 60431 Frankfurt, Germany

**Keywords:** CFTR, *CFTR* modulators, cystic fibrosis, inflammation, phagocytes

## Abstract

Cystic fibrosis (CF), the most common genetically inherited disease in Caucasian populations, is a multi-systemic life-threatening autosomal recessive disorder caused by mutations in the cystic fibrosis transmembrane conductance regulator (*CFTR*) gene. In 2012, the arrival of *CFTR* modulators (potentiators, correctors, amplifiers, stabilizers, and read-through agents) revolutionized the therapeutic approach to CF. In this review, we examined the physiopathological mechanism of chronic dysregulated innate immune response in the lungs of CF patients with pulmonary involvement with particular reference to phagocytes, critically analyzing the role of *CFTR* modulators in influencing and eventually restoring their function. Our literature review highlighted that the role of *CFTR* in the lungs is crucial not only for the epithelial function but also for host defense, with particular reference to phagocytes. In macrophages and neutrophils, the *CFTR* dysfunction compromises both the intricate process of phagocytosis and the mechanisms of initiation and control of inflammation which then reverberates on the epithelial environment already burdened by the chronic colonization of pathogens leading to irreversible tissue damage. In this context, investigating the impact of *CFTR* modulators on phagocytic functions is therefore crucial not only for explaining the underlying mechanisms of pleiotropic effects of these molecules but also to better understand the physiopathological basis of this disease, still partly unexplored, and to develop new complementary or alternative therapeutic approaches.

## 1. Introduction

Cystic fibrosis (CF), the most common genetically inherited disease in Caucasian populations, is a multi-systemic life-threatening autosomal recessive disorder caused by mutations in the cystic fibrosis transmembrane conductance regulator (*CFTR*) gene on the long arm of chromosome 7 [1]. This gene encodes for the CFTR protein, a cyclic adenosine monophosphate (cAMP)-regulated channel classically responsible for chloride (Cl^−^) and bicarbonate (HCO_3_^−^) passive transport across epithelial surfaces, crucial in maintaining epithelial surface hydration and regulating luminal pH, in turn fundamental for epithelial barrier function and innate defense [1,2]. A defective *CFTR* function strongly affects epithelial homeostasis, especially in the respiratory system, which determines much of the morbidity and mortality in CF patients, and gastrointestinal tracts.

In the lungs of people with CF (PWCF), the impaired ion transport derived from the *CFTR* dysfunction results in the dehydration and hypersecretion of mucus that contributes to airway obstruction and chronic bacterial colonization with recurrent pulmonary exacerbations, especially by *Pseudomonas aeruginosa*; furthermore, the exaggerated inflammatory response, hallmark of this disease, in synergy with an impaired resolution of inflammation, leads to progressive lung damage with bronchiectasis formation, wall thickening, and lung function decline. Although recurrent respiratory exacerbations represent a considerable stimulus for the promotion of the chronic inflammatory state that characterizes CF, increasing evidence suggests that *CFTR* dysfunction itself drives a dysregulated inflammatory response and that, before any infection, CF airways are already a perfect milieu for the amplification of the immune-inflammatory cascade [3]. The aforementioned alterations are present since early childhood and are accompanied by increasing colonization, first dominated by *Haemophilus influenzae* and *Staphylococcus aureus* and then by *P. aeruginosa*, and to a lesser extent *Burkholderia cepacia* complex (BCC) and *Stenotrophomonas maltophilia* [4,5].

Regarding the gastrointestinal system, meconium ileus at birth, distal intestinal obstruction syndrome (DIOS), and constipation are an interrelated group of obstruction syndromes with variable severity that characterize PWCF [6].

In CF, pulmonary and gastrointestinal complications are also associated with pancreatic insufficiency, diabetes, malabsorption with malnutrition, liver disease, and infertility [7]. Furthermore, an altered microbiome plays a role in disease progression, as demonstrated by the consequences of profound dysbiosis, both pulmonary and intestinal, that characterize this disease [8,9,10].

The recent evidence of the expression of *CFTR* in immune cells, combined with the well-known remarkable neutrophilic infiltration of the lungs of PWCF, sparked new studies on the role of phagocytes in the chronic inflammatory and impaired immune response that characterize CF, shifting the perspective from a rather epithelium-centric vision of this disease to a wider scenario [3,11,12,13,14,15].

In 2012, the arrival of *CFTR* modulators (potentiators, correctors, amplifiers, stabilizers, and read-through agents) has revolutionized the therapeutic approach to CF especially with the introduction of triple combination therapy (elexacaftor/tezacaftor/ivacaftor [ELX/TEZ/IVA], Trikafta and Kaftrio in United States and Europe, respectively) in 2019. This therapy, known also as highly effective modulator therapy (HEMT), realized a real paradigm shift in the management of CF as these molecules target the upstream underlying defect of the disease permitting the treatment of patients with almost one copy of F508del-*CFTR*, the most common CF-causing mutation (found in ~90% of PWCF) [2,11]. Although HEMT allows the achievement of notable clinical improvements, it does not prevent disease progression; moreover, its impact on the immune-inflammatory response is not clear [11]. In this review, we examined the physiopathological mechanism of chronic dysregulated innate immune response in lungs of PWCF with particular reference to phagocytes, critically analyzing the role of CFTR modulators in influencing and eventually restoring their function. We review Pubmed literature published from January 2012 to July 2022, using the keywords “cystic fibrosis”, “CFTR modulators”, “ivacaftor”, “lumacaftor”, “tezacaftor”, “elexacaftor”, “immune response”, “phagocytes”, “macrophages”, “monocytes”, “neutrophils”, and “inflammation”. We screened 305 articles and abstracts and then we focused on reviews, meta-analysis, and original articles including preclinical studies and randomized controlled trials (RCTs). In addition, background manuscripts published before 2012 have been included when they were useful for the clarification of the topic.

## 2. The Role of *CFTR* Dysfunction in Airway Epithelial Cells

In the airway epithelial cells (AECs), the *CFTR* plays a crucial role in maintaining ion and redox balance at the cell membrane both directly and indirectly through the modulation of the activity of other channel proteins. Specifically, its activity maintains the airway surface liquid (ASL) homeostasis which plays a key role in mucus clearance and lung defense. On the apical surface, *CFTR* drives Cl^−^ and HCO_3_^−^ secretion and regulates Na^+^ absorption by inhibiting the epithelial Na^+^ channel (ENaC) [1,16]. In case of impaired *CFTR* function, the combination of decreased Cl^−^ secretion through *CFTR* and increased Na^+^ resorption through ENaC with consequent water compartmental shifts reduces the amount of ASL decreasing the height of the periciliary fluid and impairing the mucociliary clearance (MCC) [1,11,16,17,18,19,20]. The lack of HCO_3_^−^ secretion leads to a reduced ASL pH with consequent further Na^+^ and fluid absorption because of the paucity of inhibition of ENaC by the secreted protein short palate lung and nasal epithelial clone 1 (SPLUNC1). Moreover, these processes are aggravated by the action of the non-gastric H^+^/K^+^ adenosine triphosphatase (ATP12A) that, in case of dysfunctional *CFTR*, secrets H^+^ unchecked increasing further the acidification of ASL [18].

As suggested by Rehman et al., inflammation is also a key regulator of HCO_3_^−^ secretion in CF airways and, compared with non-CF ASL, for similar levels of inflammation, the pH of CF ASL is more acidic [21]. The reduced ASL pH impairs respiratory host defense creating an unfavorable environment for the function of many antimicrobial factors such as SPLUNC1, which is also an antimicrobial protein with the capability to regulate other antimicrobial peptides (AMPs) [1,16]. In addition, *CFTR* transports glutathione and thiocyanate; the first one is a peptide with antioxidant activity mainly against reactive oxygen species (ROS) produced by immune cells in the lungs, whereas the second one is an anion required to form the potent microbicidal agent hypothiocyanous acid (HOSCN). Their lack disrupts the redox balance in airways leaving the effect of neutrophil elastase (NE), oxidants, and peroxidases out of control [4].

In the lungs of PWCF, the combination of these factors leads to the production of viscous, acidic, and mucopurulent secretions with mucus stasis, persistent airways infections, unresolved inflammation, and structural damage with progressive loss in lung function [1,16]. The airway epithelium does not passively undergo changes induced by *CFTR* dysfunction but plays a key role in determining the pathophysiology of CF, characterized by neutrophil-dominated inflammation, ineffective resolution of infection, and irreversible and progressive airway damage. Indeed, through different mechanisms, it recognizes and discriminates different microbial components and mounts a rapid protective inflammatory response [18,22,23,24].

One of the major arms of the epithelial arsenal is the toll-like receptor (TLR) that activates the NF-κB pathway leading to the expression of several proinflammatory molecules such as IL-6, IL-8, and ICAM-1. The CF airway is a milieu rich in TLR agonists (e.g., NE, *Pseudomonas* lipopeptides, LPS, and DNA) that via IL-8 signaling lead to the recruitment of neutrophils [25]. Furthermore, other NF-κB-activating pathways are the Nucleotide-Oligomerizing Domain 1 and 2 (NOD1 and NOD2), receptors expressed by AECs that detect peptidoglycan of Gram-positive or Gram-negative bacteria [26]. Interestingly, IL-17, a cytokine that has been found to be elevated in CF and implicated in the production of pro-neutrophilic mediators from CF epithelial cells by increasing IL-8 and IL-6, augments expression of NOD1, NOD2, and TLR4 priming the epithelium for a more vigorous response against pathogens recognized by these receptors [18,27].

In CF, the epithelial environment shows other peculiarities such as reduced production of NO, an essential molecule in promoting response to viral infections, due to the lack of expression of inducible NO synthase (iNOS) in CF AECs and to the oxygen deficiency variably present in CF lungs [4,28].

Finally, as described by Ghio et al., airways in CF patients are characterized by altered expression of iron-related proteins and iron accumulation; since these elements are an essential cofactor in the metabolism of many pathogens, including *P. aeruginosa* and *Burkholderia cenocepacia* that use this element for biofilm formation, adhesion, and invasion purposes, its increased availability represents a further advantage for these pathogens [29,30].

## 3. The Role of *CFTR* Dysfunction in Phagocytes: A Paradigm Shift

### 3.1. Monocyte/Macrophages

Macrophages represent the first line of defense against pathogens in the lungs and are divided into tissue-resident and recruited (monocyte-derived macrophages [MDMs]); tissue-resident macrophages are further divided in alveolar (which reside in the airway lumen) and interstitial (located in the lung parenchyma) [31].

Alveolar macrophages are the dominant immune cell in the steady state and conduct several functions: ensure and modulate the inflammatory response, either directly or by the crosstalk with the adaptive immune system, actualize the bacterial killing through a complex mechanism called phagocytosis, maintain tissue homeostasis acting as scavenger cells, and participate in regeneration processes [32,33]. The ability to promote all these functions is due to their plasticity. In 2000, Mills et al. distinguished macrophages in M1/kill and M2/repair profile on the basis of the pathway they utilize to metabolize arginine; specifically, M1 metabolize arginine to nitric oxide, an inhibitor of proliferation, whereas M2 produce ornithine, a promoter of proliferation. Macrophages are able to switch between classes, and such plasticity is important in the macrophage’s ability to regulate the acute inflammatory response [34,35].

Whether macrophages display an M1 or M2 phenotype is dependent upon the tissue milieu. M1 responses are associated with IL-6, IL-8, IL-12, and TNF-α production and cell surface expression of CD80 or CD86, which attract neutrophils and stimulate Th1 responses and further M1-type activation. On the contrary, M2 responses are linked with TGF-β, VEGF, EGF, IL-4, and IL-13 production, cell surface expression of CD163 or CD206, and the propensity to stimulate Th2 responses such as antibody production and further amplification of M2-type activation. Appropriate activation of the inflammatory response and subsequent resolution requires a balance between macrophage subpopulations [33,35,36].

Despite the clear predominance of neutrophilic infiltration, in the airways of PWCF, the absolute number of macrophages is not only increased during lung exacerbations but even from the later stages of fetal development and in young children with CF without detectable infection [37,38,39]. Furthermore, as demonstrated by Meyer et al., CF macrophages display both an augmented M1 profile, responsible for a proinflammatory phenotype with high levels of IL-8, IL-6, and TNF-α and low levels of IL-10, and an exaggerated M2 profile that might contribute to remodeling processes and fibrotic changing typical of CF. In addition, the authors concluded that Azithromycin regulates profile shifting by inhibiting M1 polarization and favoring the M2 profile [40]. However, bronchoalveolar lavage (BAL) obtained from CF lungs contains large concentrations of TNF-α, IL-1β, IL-6, and IL-8, but little IL-10, a pattern associated with M1 polarization [35]. Tarique et al. clarified these findings, obtaining MDMs from people with CF and healthy volunteers, and demonstrating that CF M2 polarization was dysfunctional as evidenced by the decreased ability to perform endocytosis [41]. On the other hand, CF M1 macrophages showed a decreased phagocytic activity and the inability to switch to the M2 phenotype with a consequent imbalance of the immune-inflammatory cascade towards a pro-inflammatory state [41].

Yoshimura et al. in 1991 demonstrated that *CFTR* is expressed not only in epithelial cells that strongly influence the lung milieu but also in human macrophages and neutrophils [42]; moreover, in macrophages, it has been detected both at the surface and intracellularly [43]. Mutations in the *CFTR* gene can affect macrophage function with different effects based on mutation type and in different phases of phagocytosis. In CF, the impaired macrophage function is not due exclusively to the altered epithelial environment, a hallmark of the disease, but also to a direct effect on macrophages [43].

The involvement of macrophages in the creation of the proinflammatory status typical of CF was demonstrated by Bruscia et al. in their work [44]. They compared the cellularity and cytokine levels in the BAL of wild-type (WT), heterozygous (HET), and CF mice after *P. aeruginosa* LPS exposure using a murine in vivo model of inflammation. The lungs of CF mice showed a more robust neutrophil infiltration with higher levels of macrophage-derived cytokine that promote their migration (KC, MIP-2, IL-8) and survival (G-CSF) with a slower neutrophil clearance in comparison with WT lungs. Furthermore, the BAL of CF mice was characterized by higher concentrations of macrophage-derived cytokine that stimulate the acute inflammatory response (IL-1α, IL-6) and innate immunity (IL-12) and lower levels of IL-10 (a cytokine with anti-inflammatory function) in comparison with WT mice. In addition, authors found that HET mice and cells have a phenotype between WT and CF, and thus, a single allelic *CFTR* mutation is sufficient to augment proinflammatory activation in response to LPS in CF, implying *CFTR*-dependent defects in CF macrophages [44]. Moreover, the direct role of CF macrophages in exacerbating the immune-inflammatory cascade was demonstrated by transplanting the bone marrow of WT into CF mice. Indeed, authors highlighted that *CFTR*+/+ myeloid cells, including macrophages, significantly reduce the inflammatory response to *P. aeruginosa* LPS in *CFTR*−/− animals [31,44].

Bonfield et al. reached the same conclusions using an in vivo mouse model, showing that defective myeloid *CFTR* contributes to increased inflammation with elevated cytokine production, the recruitment of neutrophils, and the inability to resolve infection even in presence of functional epithelial *CFTR* [45]. However, in the case of *CFTR* dysfunction, the macrophage function is not only affected at the level of the promoted altered inflammatory response but also at the level of the phagocytosis and autophagy processes [32,46]. When a pathogen is encountered, alveolar macrophages initiate the immune response releasing cytokines and chemokines and recruiting neutrophils and monocyte-derived macrophages (MDMs) that drive inflammation; specifically, MDMs are recruited through the release of monocyte chemoattractant protein-1 (MCP-1/CCL2) by macrophages that can also impact leukocyte behavior, influencing adhesion, polarization, effector molecule secretion, autophagy, killing, and survival [32,47].

In response to pathogens, macrophages also initiate the phagocytosis process that involves several phases: (i) detection of the particle to be ingested, (ii) activation of the internalization process, (iii) formation of a specialized vacuole called a phagosome, and (iv) maturation of the phagosome to transform it into a phagolysosome [48]. Macrophages sense pathogens by recognizing pathogen-associated molecules (PAMPs), such as lipopolysaccharide (LPS), flagellin, and dsRNA, through receptors known as pattern-recognition receptors (PRRs), expressed also on immune and structural cells, including airway epithelium. Furthermore, PRRs recognize the damage-associated molecular patterns (DAMPs), cytoplasmic and nuclear components such as HMGBI, ATP, and adenosine that are released into the extracellular environment by damaged cells [31]. There are several types of PRRs, but TLRs have been studied most intensely in CF. In non-CF patients, when exposed to *P. aeruginosa*, macrophages through the TLR4 and TLR5, expressed on their surface, initiate the pro-inflammatory cascade that activates NF-κB and MAPK signaling. On the CF macrophage surface, TLR4 is increased, whereas TLR5 is decreased, leading to excessive pro-inflammatory signaling [35]. Studies suggest CF macrophages have defective bacterial killing, but the underlying mechanisms are not fully understood [35]. Di et al. in their study were the first to associate the defective *CFTR* function with failed alveolar macrophage lysosomal acidification, a crucial process for efficient bactericidal activity. Indeed, they showed that *CFTR* protein participates in phagosomal pH control and therefore its dysfunction leads to a defective killing of internalized bacteria although the retained ability to phagocytose and generate an oxidative burst [49]. Deriy et al. reached a similar conclusion finding a tight correlation between *CFTR* genotype and levels of lysosomal acidification in alveolar macrophages [50]. Nevertheless, these data were subsequently confuted by two studies that showed that phagolysosomal acidification in alveolar macrophages is not dependent on *CFTR* channel activity [51,52]. More recently, similar results were highlighted by Law et al. on MDMs using optical nanosensors, a novel means that accurately measures macrophage phagolysosomal pH, although this finding has to be confirmed on alveolar macrophages using the same technique [53].

However, a study conducted by Riazanski et al. demonstrated that drugs are able to facilitate the activity of transient receptor potential canonical-6 (TRPC6) channel potentiating phagosome acidification and the bacterial-killing of CF macrophages [54]. The major difficulty in studying the impact of a dysfunctional *CFTR* on the bacterial-killing function of CF macrophages lies in the reproducibility of the observations, strongly influenced by the sensitivity of these cells to minimal changes in the environment of culture or purification. In addition, the complex cell signaling promoted in macrophages by live bacteria is extremely difficult to reproduce with opsonized beads [31]. Furthermore, *CFTR*−/− cell lines used in some studies as a surrogate for F508del cells represent a possible bias due to the lack of unfolded protein response (UPR); indeed, F508del mutation causes misfolding of the CFTR protein triggering the UPR, involving endoplasmic reticulum (ER) stress, and NF-κB activation [55].

Some studies have focused on the killing of specific pathogens by CF macrophages. A recent study demonstrated that they have impaired lysosomal degradative capacity of *B. cenocepacia*, a highly virulent member of BCC, which resides in LC3-labeled autophagosomes but not of *Escherichia coli*, which are enclosed in vacuoles that do not acquire LC3 [56]. Regarding the impaired killing of *P. aeruginosa* by CF macrophages, both defective phagocytosis function and activation of autophagy might contribute to this phenomenon. Indeed, Caveolin-1, which mediates *P. aeruginosa* internalization, is expressed at low levels in activated CF macrophages [31,57,58].

Moreover, autophagy, a fundamental mechanism of cytoplasmic protein turnover, has been directly linked to the ineffective bactericidal function in CF macrophages. Specifically, it is defective in CF airways because of the depletion of the autophagy-related protein Beclin 1 (BECN1) with consequent accumulation of SQSTM1/p62 substrate which promotes a pro-inflammatory status sequestering misfolded ubiquitinated F508del-CFTR and anti-inflammatory proteins, such as PPARγ and IK-Bα. In their study, Ferrari et al. demonstrated that the proteostasis regulator cysteamine, which restores the function of F508del-CFTR mutant, reestablished the autophagy mechanism of CF MDMs, restoring both bacterial internalization and clearance of *P. aeruginosa* through a process that involves upregulation of BECN1 [31,32,46].

In addition to acidification defects, CF macrophage phagosomes also have alterations in NADPH oxidase assembly and subsequent ROS production based on a decreased activation of cytosolic NADPH oxidase components, such as p47phox and p40phox, crucial for complex formation. This evidence, independent of pathogens but amplified by *B. cenocepacia*, results in an impaired bacterial killing with intracellular growth of bacteria such as *B. cenocepacia*, *P. aeruginosa,* and *Mycobacterium abscessus* [31,59,60,61].

Another alteration that might influence innate response was reported by Wright et al. who reported a failed expression of scavenger receptors such as the mannose receptor (CD206) and the macrophage receptor with collagenous structure (MARCO) by CF macrophages with consequent impaired binding and internalization of unopsonized particles as well as microbes resulting in dysfunctional phagocytosis [62,63].

Finally, given the key role of macrophages in extracellular iron depletion, an impairing in their iron-related protein expression profile in CF, as showed by Hazlett et al., might contribute to the elevated iron levels found in CF sputum and BAL fluid. Specifically, in their study authors reported reduced ferroportin (Fpn) and augmented transferrin receptor 1 (TfR1) levels in CF MDMs compared to non-CF MDMs with consequent advantage for pathogens such as *P. aeruginosa*, in whose metabolism iron plays a crucial role [64].

### 3.2. Neutrophils

Polymorphonuclear neutrophils (PMNs) play a central role in host defense against microbes and are early mobilized during an inflammatory response, both infectious and noninfectious, from the bone marrow, where they mainly reside [65]. In case of infectious stimulus, once arrived at the inflammation site driven by AECs and macrophage-derived cytokine and chemokine, they produce ROS, secrete antimicrobial peptides via the degranulation mechanism, eliminate microorganisms through phagocytosis process, and trap bacteria in neutrophil extracellular traps (NETs) endowed with antimicrobial activities [35,66].

Neutrophils, besides their involvement in primary host defense, also contribute to the regulation of immune responses, including the amplification of inflammatory cascade. Indeed, they produce pro-inflammatory cytokines such as IL-1, IL-6, IL-7, IL-17, IFN-γ, and TNF-α [67]. During phagocytosis, they confine pathogens in the phagosome and release into it different bioactive agents. These molecules are divided into two categories based on their provenience: proteins synthesized in myeloid precursors during granulopoiesis and stored in granules (serine proteases [azurocidin, proteinase-3, cathepsin G, and elastase], MMP [MMP-9], MPO, defensins, and lactoferrin) and ROS, produced de novo at the time of PMN activation by the NADPH oxidase [65,68,69].

In neutrophils, granules are divided into at least four different types: (i) primary granules, also known as azurophilic granules, that are the main storage site of the most toxic mediators; (ii) secondary granules, also known as specific granules; (iii) tertiary granules, that like secondary ones contain lactoferrin and MMP-9; and (iv) secretory vesicles that contain human serum albumin. These granules are also released during the degranulation process, strictly controlled by different pathways in turn activated by receptors in the plasma membrane or by the phagosomal membrane [70]. After the elimination of the pathogen, the cessation of the inflammatory response is achieved through the apoptosis of recruited neutrophils [65]. In case of failed eradication of pathogens, neutrophils continue to release their arsenal of destructive enzymes and amplify the immune-inflammatory cascade, especially in diseases such as CF, in which the epithelial lung milieu is impaired in a proinflammatory sense.

In CF, neutrophils are the predominant immune cells infiltrating the airway mucosa and the intralumenal space of bronchioles (accounting for ~70% of the total cell count in BAL fluid) driven by IL-8 and IL-17 secretion; their load as well as the extracellular activity of the protease NE in BAL fluid correlates well with disease progression in CF patients, from infancy to adulthood [71,72,73]. In normal homeostatic conditions, neutrophils are short-lived and undergo spontaneous apoptosis to guarantee the termination of the inflammatory insult but, similarly to macrophages, are also characterized by remarkable plasticity. Indeed, although when they leave the bone marrow neutrophils have already a default pro-apoptotic program, it can be modified by stimuli received in the target tissue including IL-8 and GM-CSF, that promote their persistence and longevity [71,74,75]. IL-8, also known as CXCL8, is mainly produced by AECs and macrophages, and one of its major functions is to attract and activate neutrophils and delay their apoptosis via their IL-8 receptor (mainly CXCR2) [76,77].

The elevated levels of IL-8 that characterize CF, given its anti-apoptotic action on neutrophils, contribute to determining the remarkable neutrophilic infiltration of the CF lung. Roussel et al. demonstrated that a dysfunctional *CFTR* leads to enhanced IL-8 synthesis upon exposure to *P. aeruginosa* because of the action of multiple TLRs acting redundantly. Furthermore, the decreased level of extracellular glutathione present in CF with consequent higher sensitivity to ROS further results in an increased production of IL-8 via the function of NADPH oxidase [76,78].

**Figure 1:**
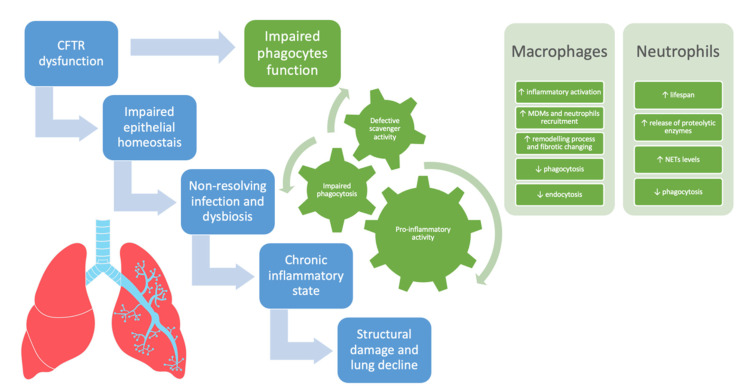
The involvement of phagocytes and neutrophils in driving lung disease progression in cystic fibrosis. CFTR = cystic fibrosis transmembrane conductance regulator, MDMs = monocyte-derived macrophages, NETs = neutrophil extracellular traps.

In CF, in addition to IL-8/IL8R derived signaling, other pathways such as the CXCR4/CXCL12 axis and the oxygen-dependent transcription factor hypoxia-inducible factor-1α (HIF-1α) might concur in prolonging the lifespan of neutrophils consequently increasing the neutrophilic infiltration [76,79,80]. Figure 1 summarizes the involvement of phagocytes and neutrophils in driving lung disease progression in CF.

Moriceau et al. theorized that the impaired apoptosis of CF neutrophils is not only linked to the chronic infectious state of CF disease, but also to CF intrinsic factors [81]. They arrived at this theory by comparing the lifespan of neutrophils of CF children with that of their parents, who were heterozygotes for the *CFTR* mutation but without chronic bacterial infection, without finding differences in the ability of PMN to undergo apoptosis, thus highlighting an innate PMN perturbation in CF. Nevertheless, in their study, they did not find a direct modulation of chloride conductance activity of *CFTR* on this programmed death mechanism suggesting that other *CFTR* functions, unknown modulatory factors, or modifying genes intrinsic to CF might be involved [81].

The common thread between the high neutrophilic infiltration that characterizes CF lungs and the recurrent lung infections is represented by the altered bactericidal activity of neutrophils, notably affected by the availability of chloride [82]. In detail, chloride is pivotal to modulating the phagosomal proteolytic activity of multiple serine proteases (proteinase 3, cathepsin G, NE); furthermore, it influences the phagosomal pH, in turn, a determinant for optimal activity of hydrolytic and proteolytic enzymes and for the formation of hypochlorous acid (HOCl), a potent phagosomal microbicide [73,82]. As stated so far, it is not surprising that when bathed in a chloride-rich buffer, normal neutrophils kill *P. aeruginosa* twofold faster than they do in a chloride-free buffer [65]. The *CFTR* channel, present in phagosomal membranes, is crucial for the chloride influx from the cytoplasm into the phagosomes; thereby a *CFTR* mutation results in impaired chlorination and killing of phagocytosed pathogens, especially those that require relatively high levels of HOCl to eradicate, such as *P. aeruginosa*, translating into a survival advantage [65,83,84,85].

The impaired microbial killing of neutrophils is also linked to intracellular calcium (Ca^2+^) homeostasis dysregulations. Robledo-Avila et al. in their study suggested that, in CF neutrophils, the increased intracellular concentrations of Ca^2+^ may be affected by *CFTR* malfunction through the involvement of the transient receptor potential (TRP) Ca^2+^ channels, which allow the influx of Ca^2+^ from the extracellular compartment, with consequent diminished NADPH oxidase response and impaired secretion of NETs [73,86].

Despite the impaired bactericidal function, neutrophils are responsible for the unchecked release of proteolytic enzymes with destructive implications on lung architecture. A major product of activated neutrophils, with a critical role in CF physiopathology, is the serine protease NE, a pivotal contributor to the structural damage of airway walls (hydrolyzing many proteins in addition to elastin), implicated also in the mucin hypersecretion, impaired MCC, inhibition of several innate immune functions by digesting opsonins and opsonin receptors, airway inflammation, and impaired macrophage phagocytosis [65,87]. Nevertheless, it plays a fundamental role in host defense underlined by the evidence that NE-knockout mice are highly susceptible to sepsis induced by Gram-negative bacteria [88].

In adult and pediatric CF BAL, the increased presence of active NE has been correlated with impaired structural integrity, worsening lung function, and decreased body mass index over time. In a study conducted by Sly et al. on CF children, the free NE in BAL fluid at 3 months of age was associated with increased odds of persistent bronchiectasis; the odds were seven times as high at 12 months of age and four times as high at 3 years of age [71,89]. In another study, conducted by Sagel et al., the NE in BAL fluid of children with CF correlated inversely with forced expiratory volume in the 1st second (FEV1) [90]. Moreover, increased NE activity is responsible for *CFTR* degradation and activation of ENaC with subsequent further aggravation of electrolyte imbalances typical of CF [87,91,92].

In addition, NE modulates the function of several proteins, most notably the matrix metalloproteinase 9 (MMP9), another neutrophil protease that plays a key role in CF disease progression, whose quantity and activity were found to increase in BAL fluid of PWCF. Indeed, although the production of MMP9 by peripheral blood mononuclear cells (PBMCs) was found constitutively augmented in CF patients homozygous for F508del mutation because of impairment in PBMCs Ca^2+^ homeostasis, NE potentiates MMP9 activity through a direct activatory cleavage and/or the degradatory cleavage of its inhibitor, the tissue inhibitor of metalloprotease-1 (TIMP-1) with subsequent augmented collagen degradation and tissue damage [71,93,94,95,96]. The NE is also able to impair the bacterial-killing activity of neutrophils via the cleavage of the IL-8 receptor CXCR1 expressed on their surface and to repress flagellin transcription in *P.* aeruginosa, which facilitates biofilm formation [71,97,98]. Additionally, as shown by Devaney et al. in their work, NE degrades TLR4 on HECs reducing bacterial lipopolysaccharide (LPS) sensitivity and increasing inflammation [5,99].

Another mechanism that impairs bacterial killing and prolongs the lifespan of neutrophils in CF is related to resistin (RETN), an inflammatory cytokine significantly upregulated in this condition [100,101]. As demonstrated by Miller et al., this protein promotes an efficient uptake and utilization of glucose in neutrophils, fueling their pro-survival pathways. Furthermore, RETN impairs their bactericidal ability by inhibiting actin polymerization, crucial for the phagocytosis process, and ROS production, as has been observed for the pathogens *P. aeruginosa* and S. *aureus* [100,102].

Another key protein in CF physiopathology, significantly increased in this disease, is the pro-inflammatory cytokine IL-17 that regulates granulopoiesis and neutrophil recruitment; it is produced mainly by CD3+CD4+ T-helper (Th), although was also identified in other “innate-like” tissue-resident lymphocytes and in neutrophils [35,103]. In a study conducted by Taylor et al. on a cohort of F508del CF subjects, authors found elevated levels of IL-17, NE, and MMP9 in sputum at the time of pulmonary exacerbation due to *P. aeruginosa* infection with decreased levels of IL-17 IV antibiotics; furthermore IL-17 producing neutrophils were the predominant cell in sputum of that cohort during the pulmonary exacerbation and a statistically significant inverse correlation between them and FEV1 was demonstrated [75].

As mentioned above, an alternative microbicidal mechanism of neutrophils is represented by the releasing of NETs, a mesh-like network that consists of neutrophil chromatin complexed with histones, pro-inflammatory mediators, and neutrophil granule contents, in a process known as “NETosis”. This process can be activated by various pathogens and their components (e.g., LPS), antibodies and immune complexes, cytokines, and chemokines (e.g., IL-8, TNF), and is distinguished in two forms: classical or suicidal and vital NETosis, both present in CF [35,104]. However, in CF lung disease, this process has limited efficacy except for the early stages and, on the contrary, it turns out to be counterproductive; indeed, as demonstrated by Gray et al., NETs provided a proinflammatory stimulus to macrophages. Moreover, pathogens can develop escape mechanisms from NET-mediated killing such as degradation of NETs using pathogen-derived DNases or developing resistance to their microbicidal components [35,105,106].

Interestingly, Nadesalingam et al. in their study showed that hypertonic saline suppresses NETosis induced by *E. coli* LPS (0111:B4 and O128:B12), and *P. aeruginosa* and LPS-induced ROS production [107].

## 4. *CFTR* Modulators and Their Impact on Phagocytes

### 4.1. From CFTR Modulators to HEMT: A New Scenario

*CFTR* modulators are molecules that increase the amount of mature CFTR protein (correctors) or enhance channel function (potentiators) at the cell surface and until the advent of HEMT these medications were reserved, alone (ivacaftor) or in combination (lumacaftor/ivacaftor [LUM/IVA] or tezacafor/ivacaftor [TEZ/IVA]), only for precise subsets of patients [2]. In 2019, the approval of the triple combination therapy with ELX/TEZ/IVA (Trikafta^®^ in the United States and Kaftrio^®^ in Europe) radically changed the natural history of CF. In fact, it significantly improves several clinical, instrumental, and laboratory outcomes (percent predicted Forced Expiratory Volume at 1st second [ppFEV1], Lung Clearance Index (LCI), rate of pulmonary exacerbations, sweat chloride concentrations, body mass index [BMI], and Cystic Fibrosis Questionnaire-Revised [CFQ-R]) augmenting the eligible subjects. Specifically, they are eligible for HEMT in all subjects aged 6 years or older with almost one copy of F508del-*CFTR*, the most common CF-causing mutation (found in ~90% of PWCF) [7,108,109].

Although *CFTR* modulators were developed with the aim to improve *CFTR* function on epithelial cells, they have a positive impact also on weight and growth, pancreatic function, the gastrointestinal and hepatobiliary systems, sinus disease, bone disease, exercise tolerance, fertility, mental health, and immunity [110]. Relatively to the effect of *CFTR* modulators on the dysregulated immune function that characterizes CF, different studies investigated the possible implications of these molecules both on the bacterial-killing function of phagocytes and on the immune-inflammatory that they orchestrate in CF lungs [110].

### 4.2. Ivacaftor

Ivacaftor was the first *CFTR* modulator approved in 2012 by the Food and Drug Administration (FDA) and European Medical Agency (EMA) with the name of Kalydeco^®^; it belongs to the class of potentiators and increases the conductance of ions and fluid, powering the protein function. Patients eligible for it are subjects older than 4 months with certain class III (G551D, G1244E, G1349D, G178R, G551S, S1251N, S1255P, S549N, S549R) or class IV mutations (R117H), for whom this potentiator represents the benchmark therapy in absence of an F508del mutation [111,112,113].

Due to the significant impact of ivacaftor therapy in eligible patients in terms of ppFEV1, weight gain, and concentration of sweat chloride and to the presence of CFTR in monocytes, macrophages, and neutrophils, the literature has shown a growing interest in the role of ivacaftor on the activity of CF phagocytes [114,115,116,117]. One of the earliest studies on this theme was conducted by Pohl et al. in 2014 [118]. In their research conducted on CF neutrophils obtained by patients homozygous for F508del-*CFTR* mutation, authors registered a decreased and delayed activity of the GTPase Rab27a that regulates the degranulation of secondary and tertiary granules with consequent impaired microbicidal activity on *P. aeruginosa* [118]. Nevertheless, the ivacaftor treatment in patients with F508del/G551D genotype resulted in a corrected Rab27 activity with a significantly improved microbial killing via a partially restored degranulation mechanism [110,119].

Subsequently, Guerra et al. in their study demonstrated that, in subjects with non-G551D *CFTR* class III mutations, ivacaftor was able to transiently increase the hydrogen voltage-gated channel 1 (HVCN1) mRNA expression to a significant extent, with a subsequent decrease after 6 months of treatment [120]. This channel protein on one side improves the efficiency of NADPH oxidase, and therefore the ROS production by minimizing depolarization-induced self-inhibition. On the other hand, it is involved in the alkalinization of phagocytic vacuoles, generating an environment optimal for proteases (neutral proteases, cathepsin G, and elastase) function. It follows that, through increasing levels of HVCN1 mRNA, ivacaftor treatment promotes the bacterial killing function of neutrophils, although temporarily [120,121].

The impact of ivacaftor on the neutrophilic trafficking to the CF lungs was explored by White et al. [122]. In their study, authors highlighted an exaggerated adhesion of neutrophils of patients with F508del and G551D mutations due to the augmented expression of CD11b on the membrane, in turn, consequent to the reduced membrane cholesterol content. This alteration in the composition of the neutrophils membrane is not directly caused by the *CFTR* impairment but rather related to the unresolved inflammation that induces ER stress, responsible for the impairment of the Ca^2+^-dependent modulation of μ-calpain with consequent proteolysis of the membrane cholesterol trafficking protein caveolin-1 [122]. In this scenario, ivacaftor, as well as lung transplant therapy, was associated with reduced levels of circulating pro-inflammatory mediators (CXCL8, TNF-α, and CXCL7 levels) and increased caveolin-1 and membrane cholesterol, with concurrent normalized neutrophil adhesion [122].

Another study that focused attention on the role of ivacaftor in reducing the constitutive activation of neutrophils and therefore the pro-inflammatory state that characterizes CF was conducted by Bratcher and colleagues [123]. Specifically, they characterized the activation state of monocytes and neutrophils in subjects with G551D mutation over a 6-month period of treatment with this *CFTR* potentiator. Interestingly, analysis of baseline data from these subjects revealed a higher expression of active CD11b on neutrophils and of CD63 on monocytes, both markers of priming/activation, compared to healthy controls which were normalized by in vivo ivacaftor treatment [123].

Delayed neutrophil apoptosis is described in CF, although it is unclear whether this is an intrinsic neutrophil defect or a response to chronic inflammation. In their study, Gray and colleagues compared the survival of non-CF and CF neutrophils obtained from patients with at least one G551D mutation prior to and 2 days after ivacaftor therapy registering a significantly decreased survival only in CF-neutrophils [124]. Furthermore, as suggested by the authors, this effect resulted in a decreased inflammatory state secondary to the decreased formation of NETs. In addition, investigating the survival of *CFTR* PMNs from *CFTR*−/− piglets in comparison to wild-type neutrophils after exposure to pro-apoptotic stimuli, they found a prolonged survival in CF ones concluding that the decreased apoptosis was primarily related to the absence of *CFTR* [124].

The effect of ivacaftor was investigated also on monocyte/macrophage by several studies that made use of modern proteomics and transcriptomics techniques. [125,126,127,128]. Hisert et al. performed a proteomic analysis of the plasma membrane of monocytes from 12 patients with *CFTR*-G551D mutations before, 2, and 7 days after treatment with ivacaftor finding a marked increase in levels of proteins implicated in cell migration [125]. Specifically, ivacaftor augmented ENO1 and PFN1, which enhance monocyte migration across epithelia and endothelia, and ICAM3 and CORO1A, which participate in leukocyte migration. Furthermore, this potentiator reduced the monocyte proteins involved in inflammation, such as S100A9, MX1, and HLA-B, via a decrease in monocyte sensitivity to IFNγ. Because of these findings, authors speculated about the possible involvement of increased sensitivity to IFNγ of monocyte in the physiopathological mechanisms underlying the CF disease resulting in hyperactivation of IFNγ-induced inflammatory pathways and in a block of monocyte migration to the lungs [125].

An analogous investigation was subsequently performed by Pedrazzi et al., who analyzed the modifications in the proteomic profile related to restored *CFTR* activity in PBMCs isolated from CF subjects carrying residual function mutations eligible for Ivacaftor therapy after ex vivo treatment with this potentiator [126]. Authors reported a downregulation both of proteins involved in the leukocyte transendothelial migration, contrary to what was highlighted by Hisert and colleagues, and in the regulation of actin cytoskeleton pathways after ivacaftor treatment. Furthermore, authors focused their attention on MMP9, whose expression was downregulated by ivacaftor, with theoretical beneficial effects on disease progression [125,126].

Another similar study was recently conducted by Hoppe and colleagues who analyzed inflammatory and growth-related proteins at baseline, 1, and 6 months after ivacaftor initiation in 64 CF subjects with G551D mutation [127]. After 1 month of treatment authors reported both significant reductions in high mobility group box-1 protein (HMGB-1), calprotectin, serum amyloid A (SAA), and granulocyte colony-stimulating factor (G-CSF), markers of inflammation, and an increase in insulin-like growth factor (IGF-1), implicated in linear growth; moreover, through 6 months of therapy, the decreased levels of HMGB-1 and calprotectin were confirmed. Furthermore, an additional analysis reported a significant change in levels of a protein involved in lipid digestion and transport and extracellular matrix organization biological processes (albumin, afamin, leptin, trypsin, pancreatic stone protein [PSP], pituitary adenylate cyclase-activating polypeptide-38, repulsive guidance molecule A [RGMA], calreticulin, GTPase KRas) after 6 months of treatment. This proteomic analysis correlates ivacaftor therapy with reduced inflammation, ameliorated lipid metabolism, improved lung function, and weight gain [128].

The aforementioned data regarding the impact of ivacaftor on inflammation and lipid metabolism are not surprising after the findings previously reported by O’Connor and colleagues [129]. Indeed, after the analysis of plasma fatty acid levels and urine prostaglandin E metabolites (PGE-M) before and after 6 months of ivacaftor therapy in 40 subjects with G551D mutation, a reduction in inflammatory PGE without a full correction of the plasma fatty acid abnormalities typical of CF was reported [129].

Similarly to the proteomics studies above mentioned, Hisert and colleagues used transcriptomics techniques to compare ivacaftor-related changes in monocyte mRNA of PBMCs obtained by PWCF with R117H mutation, a relatively common class IV CFTR mutation characterized primarily by reduced channel gating and by conductance deficit [128,130]. Their analysis showed unexpected findings since, despite clinically ivacaftor therapy being associated with diminished markers of inflammation, it upregulated 42 genes coding for canonical inflammatory cytokines, such as TNF and IL-αβ, and increased plasma levels of CXCL2 and CCL2, chemokines that recruit neutrophils and monocytes, respectively [129]. Despite the intricate interconnections between the various factors involved in the transcriptional response to ivacaftor in monocytes, globally the most involved pathways were those involving TNF signaling via NF-κB, IFNγ, IFNα, inflammatory response, cytokine signaling, and response to the bacterium. Based on the obtained results and considering the conflicting findings of the aforementioned proteomics studies, authors speculated that, although in innate immune cells a *CFTR* dysfunction predisposes to a pro-inflammatory status, in vivo the CF plasma milieu is able to override the inflammatory balance leading to an immune-suppressed (or tolerant) state. In these conditions, ivacaftor would exert both a pro-inflammatory and anti-inflammatory action on CF immune cells [125,126,127,128,131].

Zhang et al. investigated the role of ivacaftor on the bacterial-killing function of MDMs [132]. Firstly, in basal conditions, MDMs from people with CF and ivacaftor-sensitive *CFTR* variants showed decreased *CFTR* expression, increased apoptosis, and decreased phagocytosis in comparison to non-CF MDMs. Conversely, CF patients taking ivacaftor demonstrated the restoration of phagocytosis resulting in differentially enhanced macrophage-mediated bacterial killing; indeed, there was a significant 89% decrease in *P.* aeruginosa bacterial load whereas no differences in methicillin-resistant *S. aureus* (MRSA) or *B. cenocepacia* bacterial load were registered [132]. Furthermore, *CFTR* expression increased, and apoptosis decreased in CF MDMs in response to ivacaftor that was also able to improve M1 but not M2 CF macrophage polarization responses and decrease CF macrophage-derived pro-inflammatory cytokine (IL-6, TNF-α, and IL-12) to levels comparable to non-CF MDMs [132].

### 4.3. Lumacaftor + Ivacaftor

Lumacaftor was the first approved *CFTR* corrector active on F508del-CFTR protein; this molecule improves the processing, trafficking, and stability of the full-length protein leading to the increased amount of mature *CFTR* at the cell surface [133]. In 2015, its combination with ivacaftor was approved under the name of Orkambi^®^ by FDA and EMA, actually indicated for patients aged 2 years and older homozygous for F508del-*CFTR* mutation [134,135]. In a study conducted by Barnaby et al., the effect of lumacaftor alone and in combination with ivacaftor on the ability of F508del/F508del MDMs to phagocytose and kill *P. aeruginosa* was examined [136]. Surprisingly, the corrector alone restored the phagocytosis and the killing of this pathogen by CF MDMs to wild-type levels whereas the addition of ivacaftor reduced the efficacy of lumacaftor. Conversely, lumacaftor alone had no significant effect on cytokine secretion by CF MDMs whereas ivacaftor alone or in combination with lumacaftor significantly reduced the secretion of several proinflammatory cytokines, such as IL-6, IL-8, TNF-α, IFN-γ, and GM-CSF, in response to *P. aeruginosa* [136]. The subsequent study conducted by Zhang and colleagues confirmed these findings since the combination of LUM/IVA not only was unable to improve phagocytosis of F508del/F508del MDMs but even worsened it; in fact, CF MDMs on LUM/IVA had decreased *B. cenocepacia* phagocytosis compared to CF MDMs not on CFTR modulators and non-CF MDMs [132].

The anti-inflammatory properties of the LUM/IVA combination were investigated by Jarosz-Griffiths et al., who highlighted a downregulation of the nucleotide-binding oligomerization domain, leucine-rich repeat-containing protein 3 (NLRP3)-inflammasome, a key regulator of inflammation in CF. In fact, in their study authors registered a significant reduction in serum and NLRP3-stimulated IL-18 levels, TNF secretion, and caspase-1 activity and an increase in serum IL-10, a potent anti-inflammatory cytokine, by F508del/F508del LPS/ATP-stimulated peripheral blood mononuclear cells (PBMCs) exposed to LUM/IVA [137].

The transcriptional response to LUM/IVA was examined by Kopp et al., who performed a whole-blood transcriptomic analysis in 20 CF patients with F508del/F508del genotype pre- and 6 months post-LUM/IVA initiation and in 20 non-CF healthy controls [138]. Pre- and post-treatment profiles showed a differential expression of, respectively, 491 and 191 genes between the CF and non-CF groups. Specifically, the CF group profile was associated with marked overexpression of inflammation-related and apoptosis genes, including the macrophage-mediated inflammatory signaling of IL-1β/IL-18 axis, and significant underexpression of T-cell and NK cell-related genes compared to the non-CF group that persisted despite LUM/IVA therapy. However, MMP-9 levels, typically over-expressed in CF people, were reduced 1.8-fold post-LUM/IVA initiation in clinical responders, characterized by a combined increase in BMI (≥0.3) and FEV1 (≥3%). Furthermore, although the modest overall changes in gene expression post-LUM/IVA treatment, select pathways including eIF2 signaling, oxidative phosphorylation, mitochondrial dysfunction, and IL-17 signaling resulted significantly influenced by *CFTR* modulator therapy [138].

In another study conducted by Currie et al., authors demonstrated that ivacaftor, lumacaftor, and their combination were able to downregulate the exaggerated ROS production typical of human CF phagocytes in response to *Aspergillus fumigatus* without compromising their fungal killing ability; moreover, they showed that the reduced ROS production was statistically significant in PBMCs and PMNs of F508del/F508del subjects treated with ivacaftor/lumacaftor. Notably, this effect was not only limited to the CF population but was also present in non-CF people [139].

Finally, Hazlett and colleagues in their study reported that ex vivo LUM/IVA treatment in F508del/F508del MDMs lead to a reduced expression of basal TfR1 and after LPS-activation to a significantly improved Fpn expression with consequent ameliorated MDMs iron sequestration [64]. The corrected dysfunctional iron metabolism by *CFTR* modulators was associated with a diminished *P. aeruginosa* biofilm formation secondary to a reduced total iron in conditioned media [64].

### 4.4. Tezacaftor + Ivacaftor

Tezacaftor is another first-generation *CFTR* corrector developed after lumacaftor; similarly to the latter, it facilitates the intracellular processing and trafficking of normal *CFTR* gene and multiple mutant *CFTR* forms (including F508del), thereby increasing the amount of CFTR protein at the cell surface and resulting in an enhanced chloride transport with improved pharmacokinetics and fewer side effects [7,133]. In 2018, its combination with ivacaftor was approved under the name of Symkevi^®^ and Symdeko^®^ by EMA and FDA, respectively, and at present, this combination is indicated in subjects aged 6 years and older, homozygous for the F508del mutation or heterozygous for the F508del mutation with one of the mutations indicated in Table 1 [140,141].

In a recent study conducted by Badr et al., the TEZ/IVA combination was able to improve autophagy activity, lysosomal acidification and function, and bacterial clearance of F508del/F508del MDMs [56]. However, authors highlighted that these CFTR modulators selectively improved the clearance of bacteria that inhabit autophagosomes (e.g., *B. cenocepacia*) but not others that do not acquire autophagy markers (e.g., *E. coli*) [56].

The aforementioned study of Jarosz-Griffiths et al., in addition to LUM/IVA, investigated the TEZ/IVA combination effect on F508del/F508del LPS/ATP-stimulated PBMCs, revealing a similar downregulation of the NLRP3-inflammasome with consequent diminished IL-18 levels, TNF secretion, and caspase-1 activity and augmented serum levels of IL-10 in comparison to LUM/IVA [137]. Nevertheless, TEZ/IVA was also able to significantly reduce serum levels of IL-1β, typically elevated in CF and with various implications in infection and inflammation mechanisms [137].

The TEZ/IVA combination also demonstrated the potential to improve the effects of other experimental molecules. In fact, in the study of Shrestha and colleagues, these *CFTR*-associated modulators were able to improve the capability of (R)-roscovitine (seliciclib), a synthetic, low molecular weight, tri-substituted purine, to enhance the bacterial-killing function of MDMs infected with CF clinical isolates of *B. cenocepacia* and *P. aeruginosa* [142].

### 4.5. Elexacaftor + Tezacaftor + Ivacaftor

Elexacaftor is a next-generation *CFTR* corrector that facilitates the trafficking to the cell surface of mature CFTR proteins with a different mechanism as compared to first-generation correctors (e.g., lumacaftor, tezacaftor) producing a synergistic effect when in combination with them [143]. Recently, Shaughnessy et al. demonstrated that this molecule is also an effective potentiator of normal *CFTR* and the most common pathogenic *CFTR* variants across mutation Classes II, III, and IV [144].

Elexacaftor was approved in a combination regimen with tezacaftor and ivacaftor by the FDA and EMA in October 2019 and in August 2020 under the name of Trikafta^®^ and Kaftrio^®^, respectively, and it is now approved in patients aged 6 years and older who have at least one F508del *CFTR* mutation [108,109].

Although the astonishing clinical results obtained by this combination therapy in eligible patients (approximately 90% of PWCF), its impact on overall immunity and the inflammatory components remains unclear. Recently, Gabillard-Lefort and colleagues investigated the effect of ELX/TEZ/IVA on the inflammatory ATP/P2 × 7R axis in CF monocytes [145]. Firstly, they demonstrated that the effect of extracellular ATP (eATP) on NLRP3-inflammasome activation involves the P2 purinergic receptor P2 × 7R (abundantly expressed on monocytes and macrophages) and that P2 × 7R expression is regulated by *CFTR* activity, explaining, at least in part, how excessive inflammation may be directly associated with *CFTR* dysfunction. Lastly, they showed that ELX/TEZ/IVA, via the rescue of *CFTR* activity, corrected K^+^ efflux, normalized intracellular Ca^2+^, and reduced P2 × 7R expression of CF monocytes with subsequent decreased NLRP3 expression, caspase-1 activation, and IL-1β secretion upon stimulation with LPS and ATP; moreover, the HEMT normalized plasma eATP and IL-1β levels of treated individuals [145,146]. Table 2 summarizes the effects of *CFTR* modulators on phagocytes.

## 5. Conclusions

The recent introduction of *CFTR* modulators revolutionized the therapeutic approach to CF, especially since the introduction of HEMT, which has demonstrated life-changing clinical outcomes for PWCF. This literature review highlights that the role of *CFTR* in the lungs is crucial not only for epithelial function but also for host defense, with particular reference to phagocytes. In macrophages and neutrophils, the *CFTR* dysfunction compromises both the intricate process of phagocytosis and the mechanisms of initiation and control of inflammation which then reverberates on the epithelial environment already burdened by the chronic colonization of pathogens such as *P. aeruginosa* and *B. cenocepacia* ultimately leading to irreversible tissue damage. In this context, investigating the impact of *CFTR* modulators on phagocytic functions is therefore crucial not only for explaining the underlying mechanisms of pleiotropic effects of these molecules in PWCF but also to better understand the physiopathological basis of this disease, still partly unexplored, and to develop new complementary or alternative therapeutic approaches. In fact, whereas on the one hand, the analyzed studies have highlighted a partial restoration of various aspects of phagocytic functions that could explain some improved clinical outcomes, on the other hand, many aspects remain unexplored. Furthermore, although several studies evaluated the role of first-generation CFTR modulators alone or in double combination on phagocyte function, there is a lack of studies on HEMT. Therefore, it should be imperative to conduct larger and more rigorous studies that include the new combination of modulators currently available in order to more thoroughly determine their effects on phagocytes, new protagonists in the CF scenario.

## Figures and Tables

**Table 1:** Approved CFTR modulators and their indications.

Modulators	Commercial Name	Approval Year	Responsive Mutations	Approved Age
**Ivacaftor**	Kalydeco^®^ (EU/USA)	2012	G551D, S549N, G1244E, G178R, S1251N, G551S, G1349D, S1255P, R117H, E56K, K1060T, P67L, E193K, A1067T, R74W, L206W, G1069R, D110E, R347H, D579G, R1070Q, D1270N, D110H, R352Q, S945L, R1070W, R117C, A455E, S977F, F1074L, F1052V, D115H; 3849 + 10 kb C > T, 2789 + 5G > A, 327326A > G, 711 + 3A > G, E831X	≥4 months
**Lumacaftor-ivacaftor**	Orkambi^®^ (EU/USA)	2015	Two copy of F508del	≥2 years
**Tezacaftor-ivacaftor**	Symkevi^®^ (EU) Symdeko^®^ (USA)	2018	Two copy of F508del One copy of F508del in association with E56K, K1060T, P67L, E193K, A1067T, R74W, L206W, D110E, D110H, R347H, D579G, R1070Q, D1270N, R352Q, S945L, R1070W, R117C, A455E, S977F, F1074L, F1052V, D1152H, 3849 + 10 kb C > T, 2789 + 5G > A, 327326A > G, 711 + 3A > G	≥6 years
**Elexacaftor-tezacaftor-ivacaftor**	Kaftrio^®^ (EU) Trikafta^®^ (USA)	2020 (EU) 2019 (USA)	One copy of F508del	≥6 years

**Table 2:** The effects of *CFTR* modulators on phagocytes.

Study	Modulators	Cells	Effects
Pohl, 2014 [118]	IVA	Neutrophils	Partially restored degranulation mechanism via a correction of Rab27 activity
Bratcher, 2016 [123]	IVA	Monocytes and neutrophils	Reduced priming/activation via normalized levels of CD11b on neutrophils and of CD63 on monocytes
Hisert, 2016 [125]	IVA	Monocytes	Increased levels of proteins implicated in cell migration and reduced levels of proteins involved in inflammation via a decreasing in monocyte sensitivity to IFNγ
Guerra, 2017 [120]	IVA	Neutrophils	Temporarily augmented bacterial killing activity via increased HVCN1 expression
Gray, 2017 [124]	IVA	Neutrophils	Significantly decreased survival with consequent decreased inflammatory state
White, 2017 [122]	IVA	Neutrophils	Reduced levels of circulating pro-inflammatory mediators and increased caveolin-1 and membrane cholesterol with consequent normalized neutrophil adhesion
Zhang, 2018 [132]	IVA	MDMs	Improved phagocytosis and M1 polarization and decreased apoptosis, cytokine production, and *P.* *aeruginosa* bacterial burden
Hisert, 2020 [128]	IVA	Monocytes	Augmented expression of genes coding for canonical inflammatory cytokines
Pedrazzi, 2021 [126]	IVA	PBMCs	Reduced levels of MMP9 and downregulated activity of proteins involved both in the leukocyte transendothelial migration and in regulation of actin cytoskeleton pathways.
Barnaby, 2017 [136]	LUM/IVA	MDMs	Significantly reduced secretion of several proinflammatory cytokines in response to *P.* *aeruginosa*
Zhang, 2018 [132]	LUM/IVA	MDMs	Decreased *B. cenocepacia* phagocytosis compared to CF MDMs not on CFTR modulators and non-CF MDMs
Currie, 2020 [139]	LUM/IVA	PBMCs and PMNs	Significantly reduced ROS production
Hazlett, 2020 [64]	LUM/IVA	MDMs	Ameliorated iron sequestration with consequent diminished *P.* aeruginosa biofilm formation
Jarosz-Griffiths, 2020 [137]	LUM/IVA	PBMCs	Downregulated NLRP3-inflammasome activity and increased IL-10 serum levels
Jarosz-Griffiths, 2020 [137]	TEZ/IVA	PBMCs	Downregulated NLRP3-inflammasome activity, increased IL-10, and reduced IL-1β serum levels
Shrestha, 2020 [142]	TEZ/IVA	MDMs	Improved capability of (R)-roscovitine (seliciclib) to enhance the bacterial-killing function of MDMs infected *B. cenocepacia* and *P.* *aeruginosa*
Badr, 2022 [56]	TEZ/IVA	MDMs	Improved autophagy activity, lysosomal acidification and function, and bacterial clearance
Gabillard-Lefort, 2022 [145]	ELX/TEZ/IVA	Monocytes	Corrected K^+^ efflux, normalized intracellular Ca^2+^ and reduced P2 × 7R expression of CF monocytes with subsequent decreased NLRP3 expression, caspase-1 activation, and IL-1β secretion upon stimulation with LPS and ATP

Abbreviations: MDMs = monocyte-derived macrophages, PBMCs = peripheral blood mononuclear cells, PMNs = polymorphonuclear leukocytes.

## Data Availability

Not applicable for a review article.
